# Changes in initiation of adjuvant endocrine therapy for breast cancer after state health reform

**DOI:** 10.1186/s12885-021-08149-0

**Published:** 2021-05-01

**Authors:** Kirsten Y. Eom, G. J. van Londen, Jie Li, Bassam Dahman, Cathy Bradley, Lindsay M. Sabik

**Affiliations:** 1 Department of Health Policy and Management, University of Pittsburgh, 130 De Soto St, A663, Pittsburgh, PA 15261 USA; 2 Divisions of Hematology-Oncology and Geriatric Medicine, Department of Medicine, University of Pittsburgh, Pittsburgh, USA; 3 Department of Health Behavior and Policy, Virginia Commonwealth University, Richmond, USA; 4 University of Colorado Comprehensive Cancer Center, Aurora, USA

**Keywords:** Breast cancer, Adjuvant endocrine therapy, Massachusetts health reform, Health insurance, Cancer registry

## Abstract

**Background:**

Socioeconomic differences in receipt of adjuvant treatment contribute to persistent disparities in breast cancer (BCA) outcomes, including survival. Adjuvant endocrine therapy (AET) substantially reduces recurrence risk and is recommended by clinical guidelines for nearly all women with hormone receptor-positive non-metastatic BCA. However, AET use among uninsured or underinsured populations has been understudied. The health reform implemented by the US state of Massachusetts in 2006 expanded health insurance coverage and increased the scope of benefits for many with coverage. This study examines changes in the initiation of AET among BCA patients in Massachusetts after the health reform.

**Methods:**

We used Massachusetts Cancer Registry data from 2004 to 2013 for a sample of estrogen receptor (ER)-positive BCA surgical patients aged 20–64 years. We estimated multivariable regression models to assess differential changes in the likelihood initiating AET after Massachusetts health reform by area-level income, comparing women from lower- and higher-income ZIP codes in Massachusetts.

**Results:**

There was a 5-percentage point (*p*-value< 0.001) relative increase in the likelihood of initiating AET among BCA patients aged 20–64 years in low-income areas, compared to higher-income areas, after the reform. The increase was more pronounced among younger patients aged 20–49 years (7.1-percentage point increase).

**Conclusions:**

The expansion of health insurance in Massachusetts was associated with a significant relative increase in the likelihood of AET initiation among women in low-income areas compared with those in high-income areas. Our results suggest that expansions of health insurance coverage and improved access to care can increase the number of eligible patients initiating AET and may ameliorate socioeconomic disparities in BCA outcomes.

**Supplementary Information:**

The online version contains supplementary material available at 10.1186/s12885-021-08149-0.

## Background

Breast cancer (BCA) is the second most common cancer and the second leading cause of cancer death among women in the US [1]. This is also the case in the state of Massachusetts, where about 30% of new cancer cases and 13% of cancer deaths among women from 2011 to 2015 were due to BCA [2, 3]. However, due in large part to advances in adjuvant therapies, overall BCA mortality rates have steadily declined since the 1970s, and relative 5-year and 15-year survival rates among BCA patients were estimated to be 90 and 80%, respectively in 2018 [1, 4, 5]. While mortality has declined overall, gains have been greatest in affluent areas and have lagged for women in poor counties, suggesting continued sociodemographic disparities in access to care [1].

Adjuvant endocrine therapy (AET) is recommended for BCA patients with estrogen receptor (ER) or progesterone receptor (PR) positive BCA. About 60–75% of invasive BCA cases are ER-positive [4, 6], and 65% of these cancers are also PR-positive [7]. Several randomized clinical trials of tamoxifen and aromatase inhibitors (AIs) established their positive effects on overall, disease-free, and recurrence-free survival [8–18]. Given this evidence, American Society of Clinical Oncology (ASCO) and National Comprehensive Cancer Network (NCCN) guidelines recommend use of tamoxifen for BCA in premenopausal women and the use of an AI for postmenopausal women, either as primary therapy, as sequential therapy, or in the extended adjuvant setting [19, 20].

Previous research has shown that personal, social, and structural factors are associated with guideline-concordant use of AET among BCA patients [21, 22]. Older age, low socioeconomic status, non-White race/ethnicity, higher levels of comorbidities and disability, and nonprivate insurance are factors associated with delayed initiation of adjuvant BCA therapies [23–30]. In particular, health insurance coverage is a significant factor associated with initiation of guideline-recommended treatment for BCA. Multiple studies found that higher patient out-of-pocket costs were inversely associated with adherence to AETs, suggesting the importance of comprehensive insurance coverage in ensuring access to therapy along the cancer care continuum [31–33].

To better understand the impact of insurance coverage on initiation of AET and associated socioeconomic disparities among BCA patients, we examine the impacts of the Massachusetts Health Care Insurance Reform Law passed in 2006 (hereafter, Massachusetts health reform), which increased the rate of insurance coverage in the state as well as the scope of covered benefits for many who were already previously insured [34]. The reform mandated that every individual in the state have health insurance if affordable coverage is available to them. To ensure the affordability of insurance plans, the Commonwealth of Massachusetts expanded its Medicaid program (the health insurance program for low-income and disabled individuals), instituted insurance market reforms, and required employers not offering insurance to contribute to the financing of insurance premium subsidies [35]. In addition, the Commonwealth Health Insurance Connector was established to allow individuals without access to employer-sponsored insurance to purchase community-rated insurance directly [36].

This study examines changes in initiation of AET among non-elderly women in low-income areas diagnosed with BCA following health reform in Massachusetts. We hypothesize that changes in the proportion of BCA patients who initiate AET will be more substantial among women from lower-income areas, who are most likely to be impacted by the reform, than those from higher-income areas in Massachusetts.

## Methods

### Data source

Our primary data source is the Massachusetts Cancer Registry (MCR), which is administered by the Massachusetts Department of Public Health and follows standards set by the North American Association of Central Cancer Registries (NAACCR), the Commission on Cancer (CoC), the National Cancer Institute (NCI), and the Centers for Disease Control and Prevention (CDC) [37]. The MCR collects sociodemographic information of patients (age, sex, race, ethnicity, marital status, and geographic area), cancer diagnosis (date of diagnosis, primary site, stage at diagnosis, and other tumor details) and treatment information from various health care settings within the state. We also used the American Community Survey (ACS), which is administered by the US Census Bureau to collect economic and sociodemographic information from a sample of the US population [38], to estimate median household income at the ZIP-code level in 2006. The Area Health Resources Files (AHRF) were used to control for county-level health care capacity and infrastructure.

### Study population

**Fig. 1:**
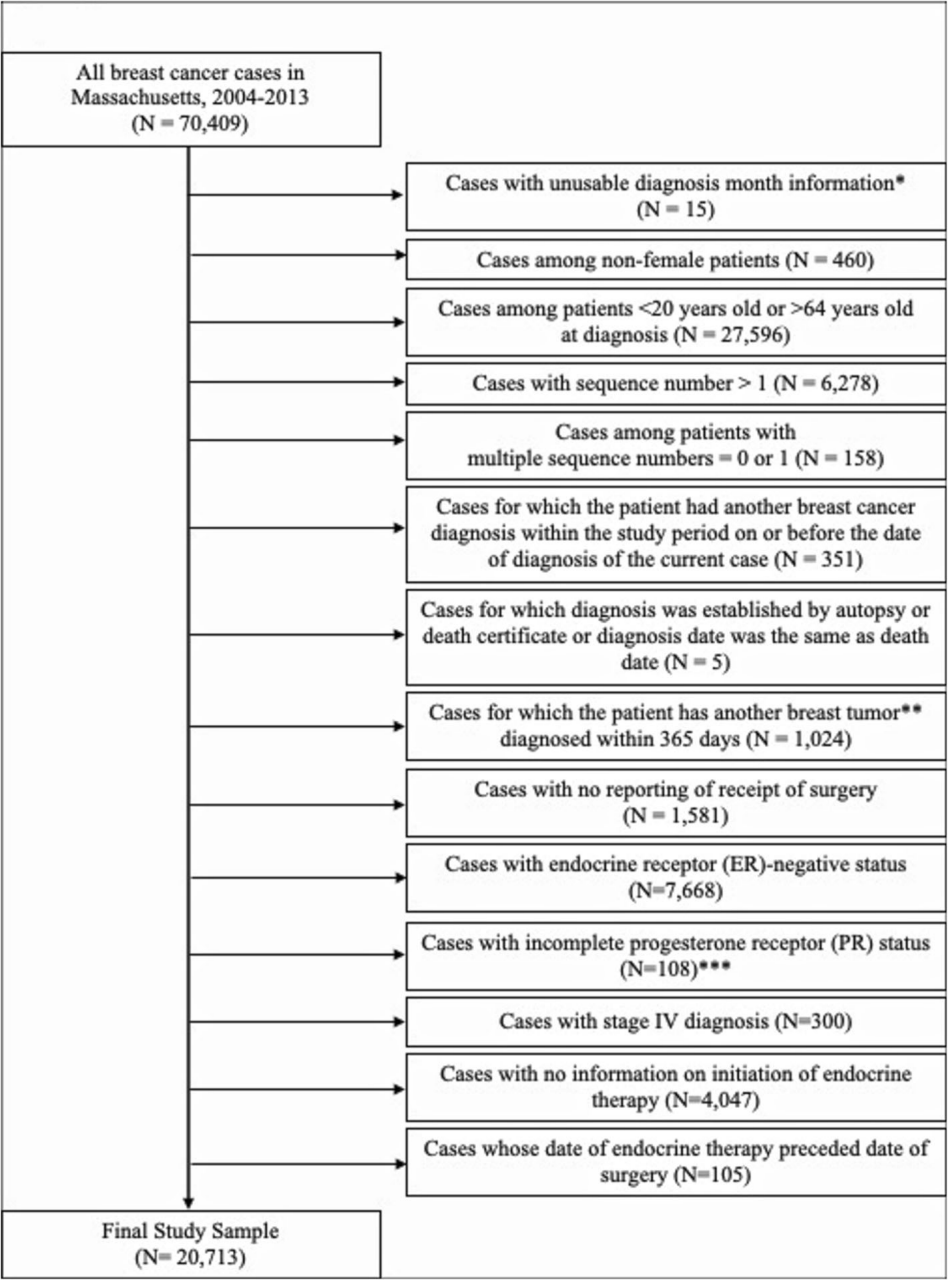
Study Sample Selection and Exclusions. *We assumed cases with missing diagnosis day but with usable data on month to have occurred on the 15th of the month. **Exclusion is based on subsequent breast cancer tumors only. ***Incomplete PR status corresponds to the following cases: PR status information was not collected for this case or not documented in patient record

Out of BCA cases diagnosed during the study period of 2004–2013 in Massachusetts, we eliminated cases among non-female patients; cases in patients under 20 or over 64 years of age, given they were not directly impacted by the insurance reforms; cases for which the patient had another BCA diagnosis within the study period on or before the date of diagnosis of the current case; cases for which diagnosis was established by autopsy or death certificate or where diagnosis date was the same as death date; cases for which the patient had another BCA diagnosed within 365 days; cases that did not receive surgery; cases with ER-negative status; cases with incomplete PR status (data not collected or not documented); cases with stage IV diagnosis; cases with no information on initiation of AET; and cases whose date of endocrine therapy preceded the date of surgery. Figure 1 illustrates how the final study sample of all ER-positive BCA surgical patients ages 20–64 years with complete AET initiation information in Massachusetts from 2004 to 2013 was derived (*n* = 20,713).

### Statistical analysis

We estimated a linear probability regression model to examine differential changes in the likelihood of initiating AET among BCA patients residing in lower-income areas compared to those in higher-income areas in Massachusetts. Our model includes a binary variable identifying lower-income ZIP code areas, another binary variable indicating the post-reform period, and an interaction term between these two binary variables to assess population-level differential impacts of the state health reform on initiation of AET among BCA patients living in lower- versus higher-income areas of Massachusetts. The outcome variable is a binary variable indicating whether the patient initiated AET after surgery. ZIP code areas with median household income below the state median value in 2006 ($68,293), were categorized as low-income areas; this designation was used to capture a population most likely impacted by the reform. The comparison group was BCA patients who lived in ZIP code areas with median household income above the state median income. The pre-reform period includes 2003–2006; the post-reform period includes 2007–2013. We adjusted for patient age, marital status, race/ethnicity, and stage at diagnosis. We controlled for secular trends in BCA treatments by adjusting for each calendar year in the model. An individual-specific error term was estimated using Huber-White robust standard errors.

We conducted two additional sets of models to test whether the changes in the initiation of AET were sensitive to definition of low-income areas and study period. The first model defined low-income areas as the lowest tertile ZIP code areas and high-income areas as the highest tertile ZIP code areas in Massachusetts and compared subgroups of patients most likely and least likely to be impacted by the health reform. The second model truncated the post-reform period in 2010 to account for the introduction of generic AIs in 2010 [32]. In addition, we estimated three additional sets of models to test robustness of results to sample inclusions and model covariates: 1) including only patients who were both ER and PR positive, 2) excluding patients whose derived American Joint Committee on Cancer (AJCC) stage was 0, and 3) adjusting for county-level characteristics including median household income, number of providers (primary care physicians, specialists, and safety net providers) per 1000 population, number of hospital beds per 1000 population, percent unemployed (> 16 years old), percent without a high school diploma (> 25 years old), percent of White Non-Hispanic/Latino, and percent urban residents [39].

To supplement our main results comparing population-level average changes in proportions of BCA patients residing in low-income areas who initiate AET after surgery to those in high-income areas, we estimated a linear model to assess overall changes in temporal trends in AET initiation. This model includes a time variable that measures the study period by quarter and ranges from 0 (first quarter of 2003) to 39 (fourth quarter of 2013), a binary variable that indicates the pre-reform period as 0 and the post-reform period as 1 which indicates the level change following the state health reform, and an interaction variable between these two variables which indicates the slope change following the state health reform. We adjusted for patient age, marital status, race/ethnicity, stage at diagnosis, and county-level characteristics described above.

## Results

Table 1 presents the characteristics of the sample before and after Massachusetts health reform. About 71% of BCA patients initiated AET statewide before the reform; after the reform, this proportion increased by 6.2 percentage points. In lower-income ZIP code areas, we observed an increase of 8.8 percentage points in the proportion of BCA patients who initiated AET after surgery; in higher-income ZIP code areas, there was an increase of 4.2 percentage points.

**Table 1:** Characteristics of the study population before and after Massachusetts health reform

	All ZIP code areas in Massachusetts		ZIP code areas below state median household income^**a**^		ZIP code areas above state median household income^**a**^	
	Pre-reform	Post-reform	Pre-reform	Post-reform	Pre-reform	Post-reform
N	*6539*	*14,174*	*2839*	*6159*	*3656*	*7897*
Adjuvant Endocrine Therapy	70.99	77.23	67.35	76.15	73.85	78.00
Stage at diagnosis^b^						
0	24.64	24.90	23.14	25.09	25.68	24.78
I	44.49	45.81	44.03	44.94	45.08	46.41
II	23.61	23.07	24.48	23.28	22.01	22.95
III	7.26	6.22	8.35	6.69	6.43	5.86
Type of Surgery						
BCS	73.93	71.89	73.09	72.74	74.07	71.15
Mastectomy with RC	9.93	17.13	8.45	14.97	11.18	18.87
Mastectomy without RC	16.15	10.98	17.86	12.29	14.85	9.98
Hormone Receptor Status						
Both ER and PR Positive	84.98	86.84	85.07	86.59	84.96	87.01
ER Positive only	15.02	13.16	14.93	13.41	15.04	12.99
Demographic Information						
Age < 40	6.35	6.03	7.20	6.67	5.58	5.57
Age 40–49	34.52	31.66	31.77	29.49	36.65	33.43
Age 50–54	20.20	20.69	20.36	19.58	20.13	21.63
Age 55–59	19.97	19.92	20.43	21.58	19.64	18.59
Age 60–64	18.96	21.69	20.15	22.68	18.00	20.78
Married	64.73	64.05	56.43	55.76	71.20	70.63
Non-Hispanic White	89.88	86.91	84.04	79.12	94.42	92.91
Non-Hispanic Black	3.38	4.54	6.23	8.39	1.18	1.56
Non-Hispanic Other Race	2.95	4.30	2.71	4.51	3.12	4.18
Hispanic	3.79	4.25	7.01	7.97	1.29	1.3

Pre-reform period is January 2003 – June 2007; Post-reform period is July 2007 – December 2013

*AET* Adjuvant Endocrine Therapy, *AJCC* American Joint Committee on Cancer, *BCS* Breast Conserving Surgery, *RC* Reconstruction, *ER* Estrogen Receptor, *PR* Progesterone Receptor

^a^The estimated median household income in MA was $68,293.37 in 2006

^b^Measured by derived AJCC Stage Group

More BCA patients were diagnosed at stage 0 or I in ZIP code areas above state median household income in the pre-reform period; however, this difference was attenuated in the post-reform period. The number of BCA patients who had a mastectomy with reconstruction increased statewide in the post-reform while the number of BCA patients who had breast-conserving surgery (BCS) decreased more in higher-income ZIP code areas. Women residing in high income areas were more likely to be married and less likely to be non-Hispanic White compared to women residing in low-income areas.

Table 2 presents differential changes in the likelihood of initiating AET between BCA patients residing in low-income versus high-income areas, adjusting for patient characteristics. There was a 5-percentage point increase (*p*-value< 0.001) in the likelihood of initiating AET following the Massachusetts reform in low-income ZIP codes, relative to the increase in the higher income ZIP codes. This effect was more pronounced among younger BCA patients ages 20–49 years, for whom we observed a 7.1-percentage point (*p*-value = 0.001) relative increase in the likelihood of initiating AET relative to the comparison group.

**Table 2:** Changes in likelihood of initiating AET after state health reform in low-income areas in Massachusetts

Breast cancer patients aged 20–64 years	0.050***
	[0.024, 0.075]
N	20,551
Breast cancer patients aged 20–49 years	0.071***
	[0.029, 0.113]
N	7960
Breast cancer patients aged 50–64 years	0.036**
	[0.004, 0.068]
N	12,591

This table presents estimates from multivariable difference-in-differences regressions comparing women living in low-income ZIP code areas to those in high-income ZIP codes of Massachusetts before and after state health reform. All models control for age at diagnosis, marital status, race/ethnicity, stage at diagnosis, and type of surgery

*AET* Adjuvant Endocrine Therapy

***p-value< 0.01 **p-value< 0.05

When we shortened the post-reform period to isolate changes after health insurance expansions prior to the introduction of generic AIs (Table 3, column 1), we observed a significant relative increase in the likelihood of initiating AET among BCA patients aged 20–64 years. However, the differential effect of Massachusetts health reform on the likelihood of initiating AET among older women residing in low-income areas was no longer significant when the post-reform period was restricted to 2007–2010. Using tertiles to define the low- and high-income areas in Massachusetts (Table 3, column 2), the estimates were robust to the alternative specification of income categories. We consistently observed a significant increase in the likelihood of initiating AET among BCA patients residing in lower-income areas in MA when we restricted the study population as ER- and PR-positive BCA patients (Table 4, Column 1), excluded patients diagnosed with in situ disease (Table 4, Column 2), and adjusted for county-level demographic and health care capacity variables (Table 4, Column 3).

**Table 3:** Changes in likelihood of initiating AET after state health reform in low-income areas in Massachusetts by age group

	(1)	(2)
	Post-reform: 2007–2010	Low-income areas: lowest tertile ZIP code areas
Breast cancer patients aged 20–64 years	0.035***	0.068***
	[0.006, 0.065]	[0.038, 0.099]
N	13,906	14,185
Breast cancer patients aged 20–49 years	0.061***	0.072***
	[0.013, 0.109]	[0.022, 0.122]
N	5588	5542
Breast cancer patients aged 50–64 years	0.018	0.064***
	[−0.019, 0.055]	[0.025, 0.103]
N	8318	8643

Model (1) re-defines the post-reform period to be 2007–2010. Model (2) re-defines the low-income areas to be the lowest tertile ZIP code areas and the high-income areas to be the highest tertile ZIP code areas in Massachusetts. Both models are based on the main multivariable difference-in-differences regressions in Table 2. All models control for age at diagnosis, marital status, race/ethnicity, stage at diagnosis, and type of surgery

*AET* Adjuvant Endocrine Therapy

***p-value< 0.01 **p-value< 0.05

**Table 4:** Robustness checks on changes in initiation of AET after state health reform in low-income areas vs. high-income area

	(1)	(2)	(3)
	Breast cancer patients who are both ER and PR positive	Excluding patients with in situ disease at diagnosis^1^	Adjusting for county-level controls^2^
Breast cancer patients aged 20–64 years	0.038***	0.049***	0.051***
	[0.106, 0.065]	[0.021, 0.076]	[0.025, 0.077]
N	17,725	15,543	20,551
Breast cancer patients aged 20–49 years	0.056**	0.087***	0.068***
	[0.012, 0.100]	[0.040, 0.133]	[0.026, 0.110]
N	7110	5801	7960
Breast cancer patients aged 50–64 years	0.025	0.026	0.040**
	[−0.009, 0.060]	[−0.009, 0.061]	[0.007, 0.072]
N	10,615	9652	12,591

These robustness checks were conducted in the main multivariable difference-in-differences regressions in Table 2, which controls for age at diagnosis, marital status, race/ethnicity, stage at diagnosis, and type of surgery

*AET* Adjuvant Endocrine Therapy, *ER* Estrogen Receptor, *PR* Progesterone receptor

***p-value< 0.01 **p-value< 0.05

^a^Derived American Joint Committee on Cancer (AJCC) stage = 0

^b^Median household income, percent unemployed, percent with less than a high school education, percent non-Hispanic white, percent urban; and primary care physicians, specialist physicians, safety net provider, and hospital beds all specified as rate per 1000 population

To assess pre-trends of the outcome, we estimated event study regressions for all models and considered the joint F-test on interactions between income category and year in the pre-period to test the pre-period significance level (Additional file 1: Appendix Table 1). We observed that the pre-trends of the outcome were not significantly different between lower-income areas and higher-income areas in MA.

**Fig. 2:**
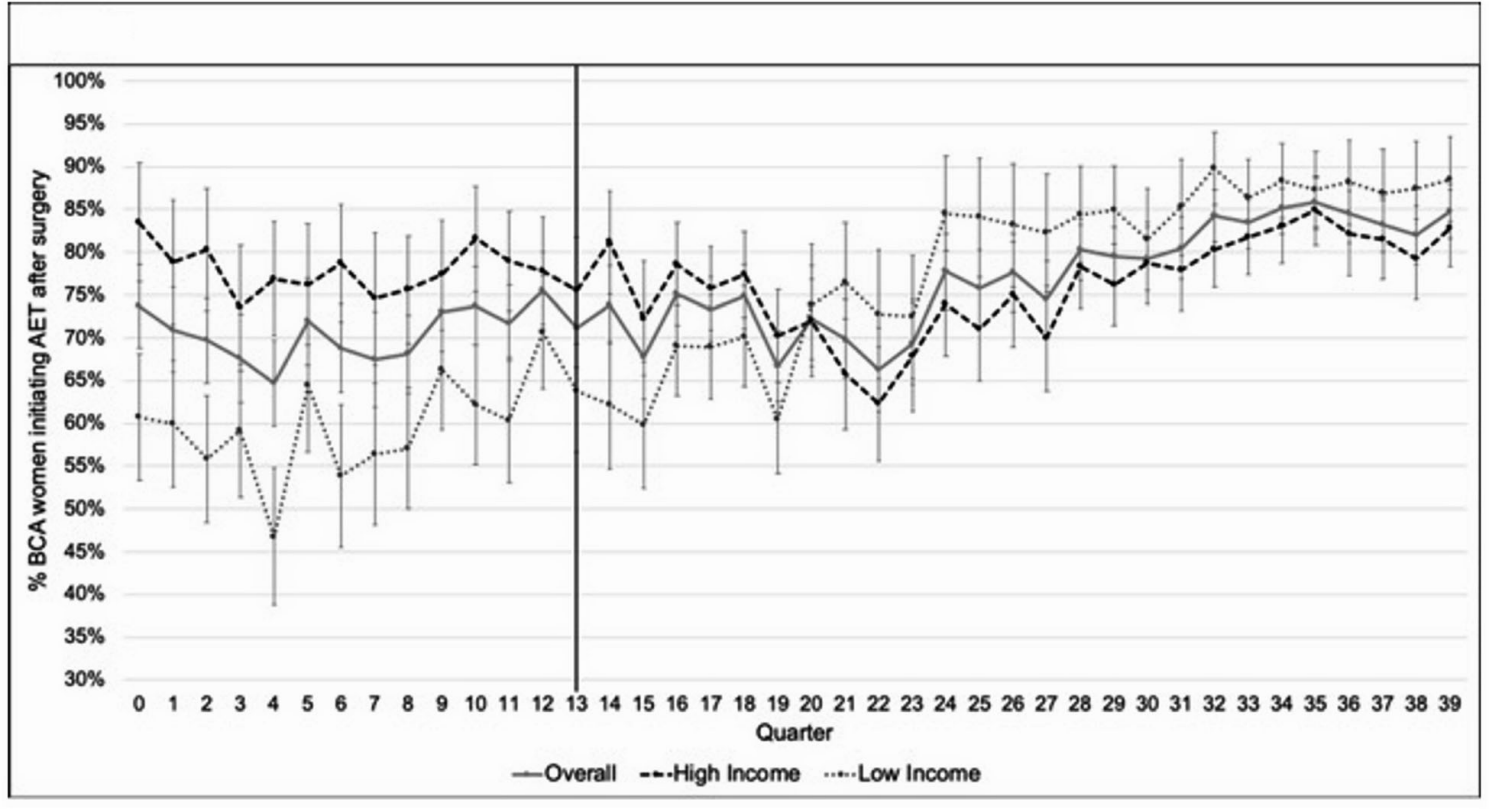
Adjusted predicted % BCA patients initiating AET in Massachusetts by quarter from 2003 to 2014. The model adjusted for age, race/ethnicity, marital status, stage at diagnosis, ER status, type of surgery received, low-, intermediate-, and high income areas, county-level characteristics including median household income, number of providers (primary care physicians, specialists, and safety net providers) per 1000 population, number of hospital beds per 1000 population, percent unemployed (> 16 years old), percent without a high school diploma (> 25 years old), percent of White Non-Hispanic/Latino, and percent urban residents. BCA: Breast Cancer; AET: Adjuvant Endocrine Therapy; ER: Estrogen Receptor

In addition, Fig. 2 presents temporal trends in the adjusted predicted percentage of BCA patients initiating AET in Massachusetts quarterly from 2004 to 2013. Considering the adjusted predicted percentage of BCA patients initiating AET in the pre-reform period, a higher percentage of BCA patients residing in high income areas in MA initiated AET after surgery than BCA patients residing in low income areas on average. However, the percentage of BCA patients residing in low income ZIP codes in MA initiating AET and that of BCA patients residing in high income ZIP codes in MA initiating AET converge at quarter 20, about 2 years after the state health reform. From quarter 20 till the end of the study period, we observe a higher proportion of BCA patients residing in low income ZIP codes in MA initiating AET after surgery than BCA patients residing in high income ZIP codes in MA.

## Discussion

In this study, we examined the differential impact of Massachusetts health reform on the initiation of AET in lower-income ZIP codes, where residents were most likely to be impacted by the reform, and higher-income ZIP codes in Massachusetts. The expansion of health insurance resulted in positive relative changes in the proportion of BCA patients in lower-income areas of the state who initiate AET. The most pronounced effects of Massachusetts health reform were in women ages 20–49 years. The relative percentage-point change between the pre-and post-reform periods among the younger sample of women was twice as large as that of older women (7.1-percentage points vs. 3.6-percentage points).

Massachusetts health reform increased access to medical care, improved financial support for safety-net hospitals, and provided more expansive prescription drug coverage. In particular, younger patients were more likely to gain coverage under state health reform. A 2004 Massachusetts Health Insurance Survey found that over 90% of newly enrolled Medicaid enrollees after Massachusetts health reform were previously unenrolled [35]. Previous studies have demonstrated that higher out of pocket prescription drug costs are associated with lower initiation and higher discontinuation of medications and treatments [27, 31, 32, 40–44]. Our study further supports these findings, estimating about a 5-percentage point relative increase in the likelihood of BCA patients aged 20–64 years in low-income areas initiating AET after reform relative to BCA patients in the same age group living in high-income areas. Given that AET is recommended for extended periods, even small monthly costs may add up to a substantial financial burden over time [45, 46].

Socioeconomic disparities in mortality among BCA patients have persisted despite increases in overall survival rates over recent decades. According to a recent report by the American Cancer Society, the mortality rate among BCA patients in poor counties was about 1.16 times higher than that in affluent counties. The observed relative increases in likelihood of initiating AET among younger women in lower-income areas who were more likely to have been without health insurance prior to the reform implies that Massachusetts health reform reduced disparities in receipt of adjuvant therapy and has important implications for health outcomes among BCA patients. This study examines initiation of AET, though AET adherence is also critical for reducing breast cancer recurrence rates, thus future studies estimating the effects of state and federal health reforms on adherence to AET would provide additional insight regarding the impact of health insurance policy changes on health outcomes.

This study has limitations. First, the limited pre-reform period might not have captured other secular trends that contributed to the increased likelihood of initiation of AET among BCA patients. Second, we did not have comparable data available from other states, limiting the geographic generalizability of our findings and the ability to include a control group that was not impacted by the reform in any way. However, this study compared ZIP codes where the median household income was below the state median household income to those where median household income was above the state median household income in an effort to compare a population most likely to be impacted by the reform to a comparable group. Given that those in higher-income areas also stood to benefit from certain provisions of the health reform, our estimates of the differential impact on patients from lower- versus higher-income areas may represent an underestimate of the full impact of reform. Fourth, due to the nature of cancer registry data, there was no relevant clinical information, including menopausal status and comorbidities, and other potential socioeconomic information including patient’s education and income that can impact initiation of AET. However, regarding patient’s menopausal status, we adjusted for BCA patients’ age as a proxy. Fifth, local area (such as ZIP code-level) health care capacity characteristics, such as the number of providers, were not adjusted for in the model. Estimates from models that adjusted for county-level health care capacity characteristics were strikingly similar to estimates from our main model.

## Conclusions

Disparities in BCA outcomes by socioeconomic factors, such as poor insurance coverage and lack of financial resources, persist. Given that about two-thirds of early-stage BCA cases are hormone-responsive, our findings indicate that expansion of health insurance coverage increases the number of eligible patients initiating AET and insurance coverage expansion may be an important policy tool for reducing income disparities in BCA outcomes, including survival. Timely initiation of and adherence to AET will result in better prognosis, which will prevent recurrence rates and improve survival of patients. This evidence from Massachusetts health reform underscores the significance of continued efforts to expand coverage across the US and emphasizes the importance of evaluating the effect of other relevant health insurance policies, such as the Affordable Care Act (ACA), on the uptake of adjuvant treatment among cancer patients.

## Supplementary Information

**Additional file 1: Table S1.** Event study estimates to test parallel trends in the pre-reform period for all models. **Table S2.** Comparing various definitions of low-income areas to estimate changes in likelihood of initiating AET after state health reform in low-income areas in Massachusetts.

## Data Availability

The datasets analyzed during the current study are not publicly available as the Massachusetts Department of Public Health accessed restricted data from Massachusetts Cancer Registry under a Data Use Agreement.

## Abbreviations

ACA: Affordable Care Act
ACS: American Community Survey
AET: Adjuvant Endocrine Therapy
AHRF: Area Health Resources Files
AI: Aromatase Inhibitor
AJCC: American Joint Committee on Cancer
ASCO: American Society of Clinical Oncology
BCA: Breast Cancer
CDC: The Centers for Disease Control and Prevention
CoC: The Commission on Cancer
ER: Estrogen Receptor
MA: Massachusetts
MCR: Massachusetts Cancer Registry
NAACCR: North American Association of Central Cancer Registries
NCCN: National Comprehensive Cancer Network
PR: Progesterone Receptor
