# Supplementary material for: Changes in initiation of adjuvant endocrine therapy for breast cancer after state health reform
Source: BMC Cancer. 2021 May 1;21:487. doi: 10.1186/s12885-021-08149-0 (PMC8088064; doi:10.1186/s12885-021-08149-0)
Supplement: Supplementary file 1 — Additional file 1: Table S1. Event study estimates to test parallel trends in the pre-reform period for all models. Table S2. Comparing various definitions of low-income areas to estimate changes in likelihood of initiating AET after state health reform in low-income areas in Massachusetts. [file 12885_2021_8149_MOESM1_ESM.docx]

# Changes in Initiation of Adjuvant Endocrine Therapy for Breast Cancer after State Health Reform

Kirsten Y. Eom^1*^, G. J. van Londen^2^, Jie Li^1^, Bassam Dahman^3^, Cathy Bradley^4^, and Lindsay M. Sabik^1^

1 Department of Health Policy and Management, University of Pittsburgh

2 Department of Medicine, Divisions of Hematology-Oncology and Geriatric Medicine, University of Pittsburgh

3 Department of Health Behavior and Policy, Virginia Commonwealth University

4 University of Colorado Comprehensive Cancer Center

***Corresponding Author**:

Department of Health Policy & Management

University of Pittsburgh Graduate School of Public Health

130 De Soto St, A663

Pittsburgh, PA 15261

Phone: (412) 277-7058

Fax: (412) 624-3146

Email: [kirsten.eom@pitt.edu](mailto:kirsten.eom@pitt.edu)

**Appendix Table 1. Event study estimates to test parallel trends in the pre-reform period for all models**

|  | **Main Model** | **Breast cancer patients who are both ER- and PR-positive** | **Breast cancer patients with in situ stage at diagnosis^1^** | **Adjusting for county-level controls^2^** |
| --- | --- | --- | --- | --- |
| N | 20,713 | 17,865 | 15,572 | 20,713 |
| Low-income ZIP*2004 | -0.085*** | -0.073*** | -0.090*** | -0.077*** |
|  | (0.019) | (0.020) | (0.020) | (0.019) |
| Low-income ZIP*2005 | -0.092*** | -0.082*** | -0.100*** | -0.079*** |
|  | (0.020) | (0.021) | (0.021) | (0.020) |
| Low-income ZIP*2007 | 0.000 | 0.003 | -0.020 | 0.016 |
|  | (0.018) | (0.019) | (0.020) | (0.019) |
| Low-income ZIP*2008 | -0.013 | -0.023 | -0.030 | -0.001 |
|  | (0.018) | (0.019) | (0.019) | (0.018) |
| Low-income ZIP*2009 | -0.053*** | -0.055*** | -0.056*** | -0.040** |
|  | (0.017) | (0.018) | (0.019) | (0.018) |
| Low-income ZIP*2010 | -0.010 | -0.002 | -0.021 | 0.004 |
|  | (0.018) | (0.019) | (0.019) | (0.018) |
| Low-income ZIP*2011 | -0.009 | -0.012 | -0.020 | 0.009 |
|  | (0.018) | (0.019) | (0.019) | (0.018) |
| Low-income ZIP*2012 | 0.015 | 0.009 | 0.022 | 0.035** |
|  | (0.017) | (0.018) | (0.018) | (0.018) |
| Low-income ZIP*2013 | -0.005 | 0.001 | -0.012 | 0.012 |
|  | (0.017) | (0.018) | (0.018) | (0.018) |
| Pre-period joint significance level | 0.808 | 0.752 | 0.744 | 0.948 |
| ^1^Derived AJCC stage = 0  ^2^Median household income, percent unemployed, percent with less than a high school education, percent non-Hispanic white, percent urban; and primary care physicians, specialist physicians, safety net provider, and hospital beds all specified as rate per 1000 population  The p-values>0.05 for this test signify that the assumption of parallel trends between the treatment and the control groups was supported.  **AET**: Adjuvant Endocrine Therapy; **ER**: Endocrine Receptor; **PR**: Progesterone receptor | | | | |

**Appendix Table 2. Comparing various definitions of low-income areas to estimate changes in likelihood of initiating AET after state health reform in low-income areas in Massachusetts**

|  | Model (1) | Model (2) | Model (3) |
| --- | --- | --- | --- |
| Breast cancer patients aged 20-64 years | 0.079** | 0.066*** | 0.050*** |
|  | [0.034, 0.123] | [0.035, 0.097] | [0.024, 0.075] |
| N | 6,505 | 13,774 | 20,551 |
| Breast cancer patients aged 20-49 years | 0.093* | 0.079** | 0.071** |
|  | [0.021, 0.165] | [0.029, 0.129] | [0.029, 0.113] |
| N | 2,550 | 5,366 | 7,960 |
| Breast cancer patients aged 50-64 years | 0.065* | 0.056** | 0.036* |
|  | [0.008, 0.122] | [0.017, 0.095] | [0.004, 0.068] |
| N | 3,955 | 8,408 | 12,591 |
| \| This table presents estimates from multivariable difference-in-differences regressions comparing women living in low-income ZIP code areas to those in high-income ZIP codes of Massachusetts before and after state health reform.  Model (1) defined low-income areas as ZIP codes whose median household income level was below the 16^th^ percentile and high-income areas as ZIP codes whose median household income level was above the 84^th^ percentile. Model (2) defined low-income areas as ZIP codes whose median household income level was below the 33^rd^ percentile and high-income areas as ZIP codes whose median household income was above the 66^th^ percentile. Model (3) defined low-income areas as ZIP codes whose median household income was below the state median ZIP code-level household income and high-income areas as ZIP codes whose median household income was above the state median ZIP code-level household income. All models control for age at diagnosis, marital status, race/ethnicity, stage at diagnosis, and type of surgery.  ***p-value<0.001 **p-value<0.01 *p-value<0.05 \| \| --- \| | | | |
